# Doxorubicin-Loaded Tumor-Targeting Peptide-Decorated Polypeptide Nanoparticles for Treating Primary Orthotopic Colon Cancer

**DOI:** 10.3389/fphar.2021.744811

**Published:** 2021-10-15

**Authors:** Guoliang Liu, Min Wang, Hongyu He, Jiannan Li

**Affiliations:** ^1^ Operating Theater and Department of Anesthesiology, The Second Hospital of Jilin University, Changchun, China; ^2^ Department of General Surgery, The Second Hospital of Jilin University, Changchun, China

**Keywords:** colorectal cancer, chemotherapy, nanoparticles, tumor targeting, drug delivery system

## Abstract

Colorectal cancer is the third most common malignant disease worldwide, and chemotherapy has been the standard treatment for colorectal cancer. However, the therapeutic effects of chemotherapy are unsatisfactory for advanced and recurrent colorectal cancers. Thus, increasing the treatment efficacy of chemotherapy in colorectal cancer is a must. In this study, doxorubicin (DOX)-loaded tumor-targeting peptide-decorated mPEG-P(Phe-co-Cys) nanoparticles were developed to treat orthotopic colon cancer in mice. The peptide VATANST (STP) can specifically bind with vimentin highly expressed on the surface of colon cancer cells, thus achieving the tumor-targeting effects. The nanoparticles are core-shell structured, which can protect the loaded DOX while passing through the blood flow and increase the circulation time. The disulfide bonds within the nanoparticles are sensitive to the glutathione-rich microenvironment of tumor tissues. Rupture of disulfide bonds of the nanoparticles leads to the continuous release of DOX, thus resulting in the apoptosis of the tumor cells. The *in vivo* experiments in mice with orthotopic colon cancer demonstrated that the synthesized DOX-loaded tumor-targeting peptide-decorated polypeptide nanoparticles showed properties of drug delivery systems and exhibited good antitumor properties. The synthesized nanoparticles show appropriate properties as one of the drug delivery systems and exhibit good antitumor properties after encapsulating DOX.

## Introduction

Colorectal cancer was the third most common malignant disease worldwide, and the number of patients was rising, with approximately 500,000 deaths each year (Jemal et al., 2011; Laroui et al., 2011). With the innovation of treatment theories, chemotherapy was highlighted. Recent advances in chemotherapy, including the use of irinotecan, oxaliplatin, fluoropyrimidines, cetuximab, bevacizumab, and radiation therapy, have increased the median survival of patients (Ortiz et al., 2012). However, advanced and recurrent colorectal cancers were still hard to be cured (Chaudhary et al., 2011). Therefore, patients with colorectal cancer needed more efficient chemotherapy (Soster et al., 2012; Prados et al., 2013).

With the development of nanotechnology, plenty of nanoscale drug delivery systems (DDSs) have been applied to the chemotherapy drug delivery, such as micelles, vesicles, nanogels, and dendrimer (Gu et al., 2015; Li et al., 2018; Chen et al., 2020; Su et al., 2020). Among them, the polymer nanoparticles were possibly the most promising (Choi et al., 2012; Wang et al., 2012; Ai et al., 2021). Through special design, nanoparticles could achieve specific functions, such as increasing the solubility of chemotherapy drugs and improving the stability in circulation (Cherukuri and Curley, 2010; Laroui et al., 2011). Nanoparticles have attracted worldwide interest and made great progress in design and fabrication.

In general, most nanoparticles possessed good biocompatibility and biosecurity (Zhu et al., 2010; Cho et al., 2012). Therefore, the loading of antitumor drugs into nanoparticles is needed to treat cancer. For the package of antitumor drugs, small-molecule drugs were promising, such as doxorubicin (DOX) and curcumin (Kaminskas et al., 2012). DOX is a kind of antitumor antibiotic with a wide antitumor spectrum (Cui et al., 2012; Maeda, 2012). Clinically, DOX could inhibit some common tumors and had been used to treat breast cancer, bladder cancer, lung cancer, ovarian cancer, colorectal cancer, and so on (Ta et al., 2009). However, the broad distribution of DOX limited the treatment efficiency and caused a variety of side effects, such as inhibition of medullary hematopoiesis function, nephrotoxicity, and cardiotoxicity (Lee et al., 2006).

In this study, a kind of mPEG-P(Phe-co-Cys) copolymers was developed, which could self-assemble into nanoparticles in aqueous solutions. The mPEG-P(Phe-co-Cys) nanoparticles (mNPs) were decorated with peptide VATANST (STP), and DOX was encapsulated within the nanoparticles to obtain STP-mNPs/DOX. STP-mNPs/DOX are core-shell structured with DOX as the core and the PEG as the shell. The appropriate particle size can prolong the circulation time while passing through the blood flow. STP could specifically bind with vimentin highly expressed on the surface of colon cancer cells (Kim et al., 2020; Vermani et al., 2020), thus increasing the tumor-targeting effects of STP-mNPs/DOX. The disulfide bonds within the nanoparticles were sensitive to the glutathione-rich microenvironment of colon cancer (GSH) (Zinczuk et al., 2020). The disulfide bonds of STP-mNPs/DOX are ruptured in the tumor tissues and led to the continuous release of loaded DOX, thus increasing the necrosis of tumor cells. The treatment efficacy of STP-mNPs/DOX in primary orthotopic colon cancer was evaluated using mice in this study.

## Materials and Methods

The preparation of mPEG-P(Phe-*co*-Cys) nanoparticles (mNPs) and STP-mNPs and characterizations of mNPs and STP-mNPs are in the Supplementary Material.

### Materials

Polyethylene glycol monomethyl ether (mPEG), L-phenylalanine (L-Phe), L-cysteine (L-Cys), and deuterated trifluoroacetic acid (TFA-*d*) were purchased from Sigma-Aldrich (Shanghai, PR China). The STP, DOX hydrochloride (DOX HCl), and GSH were obtained from Gill Biochemical Co., Ltd. (Shanghai, PR China). Cell culture products, including Dulbecco’s modified Eagle’s medium (DMEM) and fetal bovine serum (FBS), were provided by Gibco (USA). Penicillin and streptomycin were obtained from Huabei Pharmaceutical Co., Ltd. (Hebei, PR China). Sodium cyanoborohydride (NaBH3CN), 3-(4,5-dimethyl-thiazol-2-yl)-2,5-diphenyl tetrazolium bromide (MTT), and 4′,6-diamidino-2-phenylindole dihydrochloride (DAPI) were purchased from Sigma-Aldrich (Shanghai, P. China). Terminal deoxynucleotidyl transferase-mediated deoxyuridine triphosphate nick-end labeling (TUNEL) kit was purchased from Roche Company (Mannheim, Germany). The purified deionized water was prepared by the Milli-Q plus system (Millipore Co., Billerica, MA, USA).

### Preparations of mNPs/DOX and STP-mNPs/DOX

DOX was loaded into mNPs and STP-mNPs through a nanoprecipitation technique. In brief, DOX HCl was dissolved in 2.0 ml of phosphate-buffered saline (PBS) solution, and then the solution was slowly added to 18.0 ml *N*,*N*-dimethylformamide (DMF) (10.0 mg ml^−1^) containing mNPs or STP-mNPs. After that, 18.0 ml distilled water and 2.0 ml PBS were added to the mixed solution. The solution was continuously stirred at room temperature for 12 h and then dialyzed in deionized water for 12 h (molecular weight cut-off (MWCO) = 3,500 Da). At last, the mNPs/DOX and STP-mNPs/DOX were obtained by lyophilization.

### Characterizations of mNPs/DOX and STP-mNPs/DOX

The drug loading content (DLC) of mNPs/DOX and STP-mNPs/DOX was calculated using DLC (%) = (the amount of drug in the nanoparticles/total mass of the nanoparticles × 100%). The drug loading efficiency (DLE) of mNPs/DOX and STP-mNPs/DOX was calculated using DLE (%) = (the amount of drug in the nanoparticles/total amount of the drug × 100%).

The morphologies of mNPs/DOX and STP-mNPs/DOX were revealed by transmission electron microscopy (TEM) on a JEM-1011 (JEOL, Tokyo, Japan). The hydrodynamic diameters (*D*_h_) were detected by dynamic laser scattering (DLS) measurements with a scattering angle at 90° on a WyattQELS instrument (DAWN EOS, Wyatt Technology Corporation, Santa Barbara, CA, USA).

### DOX Release *In Vitro*

The DOX release profiles of mNPs/DOX and STP-mNPs/DOX *in vitro* were determined at pH 5.5, 6.8, and 7.4 with or without 10.0 mM glutathione (GSH) in PBS solution. The DOX-loaded freeze-dried micelles were dissolved into PBS at pH 5.5, 6.8, and 7.4 with or without 10.0 mM GSH, and the concentration of the solution was 100.0 μg ml^−1^, respectively. Then 10.0 ml of each solution was transferred into a dialysis bag (MWCO = 3,500 Da). Extremity-sealed dialysis bags were then placed into the homologous 100.0 ml of PBS for the release assay with 75 rpm electric shock at 37°C, which simulated the circulation *in vivo*. At pre-set times, 2.0 ml of external soaking solution was taken out and the equivalent fresh PBS of different pH with or without GSH was replenished into the homologous sample. Afterward, the accumulative DOX release was tested by fluorescence spectroscopy using the standard curve method (λ_ex_ = 480 nm).

### Cellular Uptake and Intracellular DOX Release

Murine colon cancer cell line CT26 was cultured in complete DMEM with 10% (v/v) FBS, penicillin (50.0 IU ml^−1^), and streptomycin (50.0 IU ml^−1^) at 37°C in a 5% (v/v) carbon dioxide atmosphere. The cellular uptake and intracellular release DOX profiles of DOX-loaded nanoparticles were detected by both flow cytometry (FCM) and confocal laser scanning microscopy (CLSM) using CT26 cells. Germfree coverslips were put onto 6-well plates, one for each well. The CT26 cells were seeded into the well with a density of 2.0 × 10^5^ cells per well in 2.0 ml of DMEM and cultured for 12 h. Then, three wells were chosen, and 200 μl of buthionine-sulfoximine (BSO) PBS solution was added to the selected wells with a concentration of 0.01 mmol ml^−1^. After 12 h of culture at 37°C in a 5% (v/v) carbon dioxide atmosphere, another three wells were selected, and 200 μl of GSH solution in PBS with the same concentration of BSO solution was added to the wells. After 2 h of culture, the medium was replaced with free DOX, mNPs/DOX, or STP-mNPs/DOX solution in DMEM at a final DOX concentration of 10.0 μg ml^−1^. Then, after 2 h of culture, the coverslips that were adhered with CT26 cells were washed with PBS and then fixed with 4% PBS-buffered formaldehyde for 30 min at room temperature. Subsequently, the coverslips were counterstained with DAPI (blue color) for cellular nuclei. The cellular microimages were determined on the CLSM (LSM 780, Carl Zeiss, Jena, Germany). For the FCM assay, the CT26 cells were cultured similarly to CLSM tests without any coverslips. After culture with free DOX, mNPs/DOX, or STP-mNPs/DOX for 2 h, the culture media were removed. The obtained cells were washed three times with PBS. The cells in each well were then suspended in 1.0 ml of PBS and centrifuged for 4 min at 3,000 rpm. The cells were then resuspended in 0.3 ml of PBS, and the data for 10,000 gated events were collected. The data were analyzed on a flow cytometer (Beckman, California, USA).

### Cytotoxicity Assays *In Vitro*

The cytotoxicity of free DOX, mNPs/DOX, and STP-mNPs/DOX *in vitro* in the different external environments was demonstrated using the MTT assay. The CT26 cells were seeded in 96-well plates, 7,000 cells per well, in 200.0 μl of complete DMEM and cultured for 12 h. Then, BSO solution with a concentration of 0.01 mmol ml^−1^ was added to three lists of wells. After 12 h of culture, another three lists were selected for adding GSH solution with the same concentration of BSO solution and then cultured at constant temperature for 2 h. Subsequently, the culture medium was replaced with 200.0 μl of fresh medium containing free DOX, mNPs/DOX, or STP-mNPs/DOX at a final DOX concentration of 10.0 μg ml^−1^. The cells were subjected to MTT assay after 48 h culture. The stock solution containing 0.05 mg of MTT in PBS was added to each well and then cultured for another 4 h. Subsequently, the medium was replaced with 150 μl of DMSO. Furthermore, mNPs and STP-mNPs (0–40.0 mg L^−1^) were also cultured with CT26 cells as above, but BSO or GSH solutions were not used. The absorbency of the above solution was measured on a Bio-Rad 680 microplate reader at 490 nm. The cell viability was calculated using the following: cell viability (%)= (absorbance of sample/absorbance of control × 100%).

### Pharmacokinetics of DOX *In Vivo*

For the *in vivo* research of pharmacokinetics of free DOX, mNPs/DOX, and STP-mNPs/DOX, male Kunming mice weighing about 200 g were fasted for 12 h before the test. Free DOX, mNPs/DOX, and STP-mNPs/DOX were dissolved with PBS. After intravenous administration of 5.0 mg kg^−1^ of free DOX and an equivalent dose of mNPs/DOX and STP-mNPs/DOX, blood was collected from the retrobulbar vein into heparinized 1.5 ml centrifuge tubes at 0, 5, 15, and 30 min and 1, 2, 3, 4, 6, 8, 10, 12, and 24 h. To separate the plasma and blood cells, blood samples were centrifuged at 10,000 rpm and 4°C for 10 min. The supernatant (200.0 μl) was transferred into a new centrifuge tube with 1.0 ml of methyl alcohol. After vibration by the vortex mixer for 10 min, the samples were centrifuged again. Then, the supernatants were removed into glass tubes, respectively; moreover, the samples were blow-dried using a nitrogen concentrator at 35°C. Subsequently, the samples were redissolved with 200.0 μl of methyl alcohol, and the amounts of DOX of samples were detected with the High-Performance Liquid Chromatography (HPLC) method. Waters liquid chromatographic system (Waters e2695 Separations Module, USA) was equipped with a fluorescence detector (Waters 2475 Multi-λ Fluorescence Detector, USA) with the excitation and emission wavelengths at 472 and 592 nm, respectively. A Waters Symmetry C18 analytical column (5 μm, 4.6 × 250 mm) was used at 35°C.

### *In Vivo* Antitumor Assay

The *in vivo* antitumor efficacy of free DOX, mNPs/DOX, and STP-mNPs/DOX was demonstrated using a drug-induced *in situ* tumor model in 6–8 -eek-old male Balb/C mice. Dimethyl hydrazine (DMH) was the inducer and was dissolved in ethylene diamine tetraacetic acid (EDTA) solution. EDTA was dissolved in PBS solution, and the concentration was 1 mmol L^−1^. The concentration of DMH solution was 3 mg ml^−1^. DMH solution was injected into the mice’s abdominal cavity, and the drug was absorbed through the peritoneal. The given dose was 30 mg per kilogram of body weight. The injection was performed on Tuesday and Friday every week, and the whole process lasted 16 weeks. After the last injection, one mouse was sacrificed, and the colon was dissected to confirm the formation of the tumor. After 3 days, mice were randomly divided into four groups. The tumor-bearing mice were treated with free DOX, mNPs/DOX, STP-mNPs/DOX, and PBS. The tail vein injections of 0.2 ml of PBS alone or DOX-loaded micelles of free DOX with an equivalent DOX dosage (5.0 mg DOX per kilogram body weight) in PBS were performed on days 1, 5, 9, 13, 17, and 21. Free DOX and PBS were administred as positive and negative controls, respectively.

### Histological and Immunohistochemical Analyses

Five days after the last injection, all the mice were sacrificed, and the colons were dissected along the longitudinal axis. The lining of the colons was rinsed in deionized water, flattened, and photographed. Then, the colon segments were rinsed in deionized water again, flattened, and photographed. The number of the tumor sites larger than 2 mm in each mouse was recorded. The colons and major organs (i.e., heart, liver, spleen, lung, and kidney) of other mice were collected and fixed in 4% (w/v) PBS-buffered paraformaldehyde overnight and then embedded in paraffin. The paraffin-embedded organs were cut into 5.0 μm slices. The slices were stained with hematoxylin and eosin (H&E) and cut into 0.3 μm sheets for immunohistochemical analyses (i.e., Ki-67, PARP, TUNEL). The histological and immunohistochemical alterations were detected by microscope (Nikon Eclipse *Ti*, Optical Apparatus Co., Ardmore, PA) and subsequently analyzed with CLSM and ImageJ software (National Institutes of Health, Bethesda, Maryland).

### Statistical Analyses

All experiments were performed at least three times, and the results were represented as means ± standard deviation (SD). Data were analyzed using SPSS 14.0 (SPSS Inc., Chicago, IL, USA). *p < 0.05 was considered statistically significant, and **p < 0.01 and ***p < 0.001 were considered highly significant.

## Results and Discussion

In this study, DOX-loaded tumor-targeting peptide-decorated polypeptide nanoparticles were developed for treating primary orthotopic colon cancer in mice. Vimentin was a kind of epithelial-to-mesenchymal (EMT) marker highly expressed in many cancers, including colorectal cancer (Kim et al., 2020; Vermani et al., 2020). STP could specifically bind with vimentin, which can increase the tumor-targeting effects of mNPs (Qiu et al., 2020). The STP-mNPs/DOX are core-shell structured with DOX loaded in the core, and the PEG shell can protect the inside DOX while allowing the blood flow through. The STP-mNPs/DOX is based on mPEG-P(Phe-co-Cys) copolymers, and the disulfide bonds within the nanoparticles were sensitive to the GSH-rich microenvironment of colon cancer (Wen et al., 2019; Zinczuk et al., 2019). The concentrations of GSH were 1.0–2.0 μmol/L, 2.0–20.0 μmol/L, and 2.0–10.0 mmol/L in plasma, normal tissues, and tumor cells, respectively (Schafer and Buettner, 2001). The GSH concentration in tumor cells was about 500 times higher than that in normal tissues. The rupture of disulfide bonds of STP-mNPs/DOX led to the continuous release of loaded DOX, thus increasing the necrosis of tumor cells (Wang et al., 2020; Wang et al., 2021). As a result, under the enhanced permeability and retention (EPR) and tumor-targeting effects, STP-mNPs/DOX can accumulate in tumor tissues. Large amounts of DOX can be released under high levels of GSH within the tumor cells. Furthermore, the efficacy of STP-mNPs/DOX in the treatment of primary orthotopic colon cancer was evaluated in this study.

Based on our previous study (Qiu et al., 2020), the mNPs/DOX and STP-mNPs/DOX were successfully synthesized. Supplementary Figure S1 shows the proton nuclear magnetic resonance (1H NMR) and Fourier-transform infrared (FTIR) spectroscopy of mNPs and STP-mNPs. Detailed descriptions are provided in the Supplementary Material. The DLC and DLE of mNPs/DOX are 6.42 ± 1.05 wt% and 36.12 ± 1.85 wt%, respectively. The DLC and DLE of STP-mNPs/DOX are 7.12 ± 1.85 wt% and 37.20 ± 2.55 wt%, respectively. The decoration of STP did not change the DLC or DLE of mNPs/DOX significantly.

**FIGURE 1:**
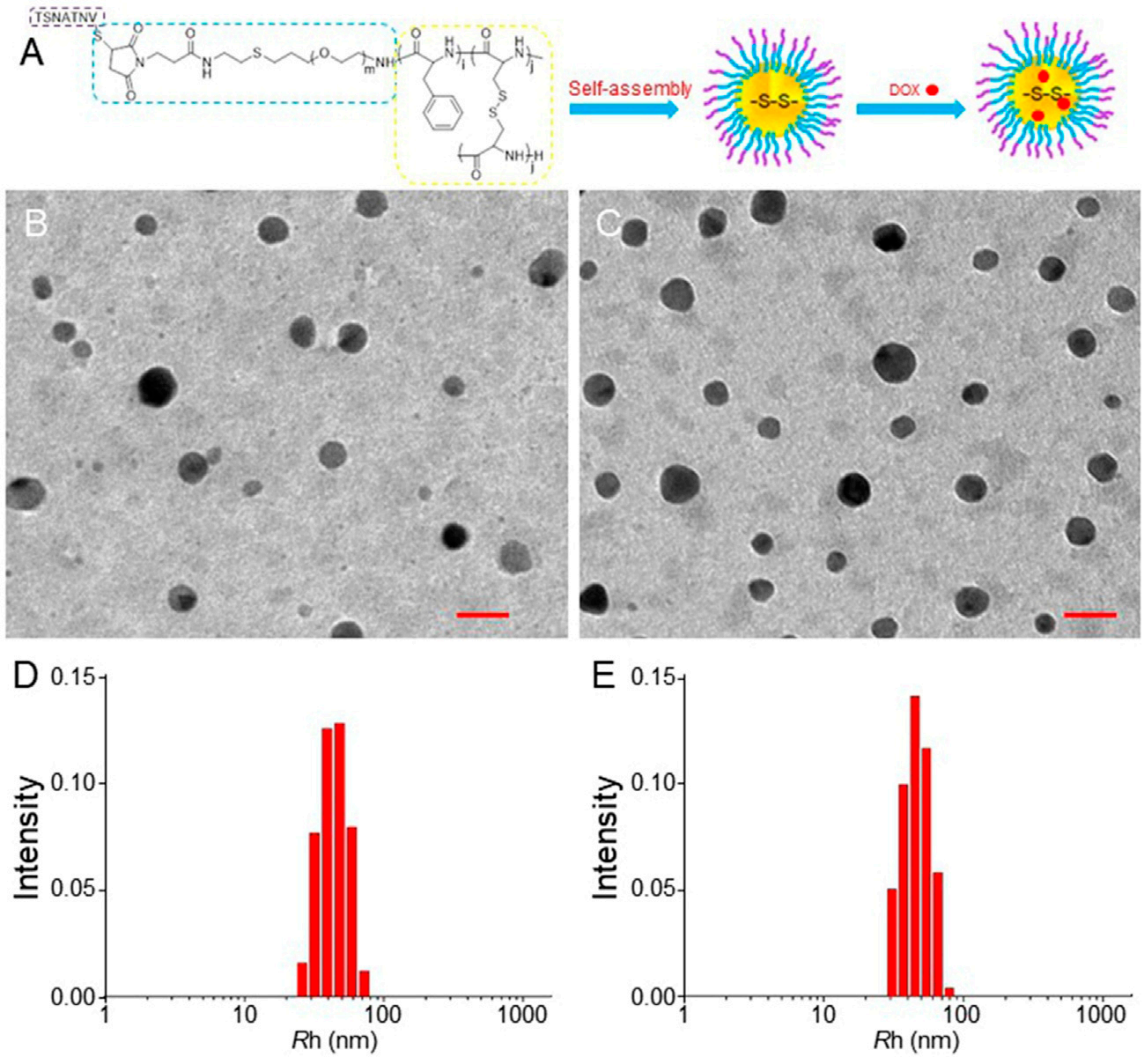
Preparation of STP-mNPs/DOX and characterizations of mNPs-DOX and STP-mNPs/DOX. **(A)** Schematic illustration of preparation of STP-mNPs/DOX. TEM analysis of **(B)** mNPs/DOX and **(C)** STP-mNPs/DOX and *R*_h_s of **(D)** mNPs/DOX and (E) STP-mNPs/DOX. Scale bars = 100 nm.

Figure 1A shows the schematic illustration of the preparation of STP-mNPs/DOX. The morphology and size of mNPs/DOX and STP-mNPs/DOX were determined by TEM and DLS. As shown in Figures 1B,C, both mNPs/DOX and STP-mNPs/DOX are spherical particles in an aqueous environment with similar sizes. DLS shows the diameters of mNPs/DOX and STP-mNPs/DOX are 88.34 ± 4.26 nm and 90.74 ± 4.65 nm, respectively (Figures 1D,E). For nanoparticles, size was a crucial property for distribution. Some studies have suggested that the particles with a radius larger than 100 nm would be captured by the reticuloendothelial system, and the particles with a radius smaller than 30 nm would be eliminated from plasma rapidly (Peer et al., 2007; Wang et al., 2012). This result indicated that the sizes of mNPs/DOX and STP-mNPs/DOX were appropriate for DDSs. Moreover, the morphology of the drug delivery systems plays an important role in the distribution within the body tissues. Spherical nanoparticles are more likely to be endocytosed within tumor cells (Qiu et al., 2020). Benefiting from the appropriate size and spherical morphology of nanoparticles, mNPs/DOX and STP-mNPs/DOX prolonged circulation and increased DOX accumulation in tumor sites through the EPR effect (Kobayashi et al., 2013).

**FIGURE 2:**
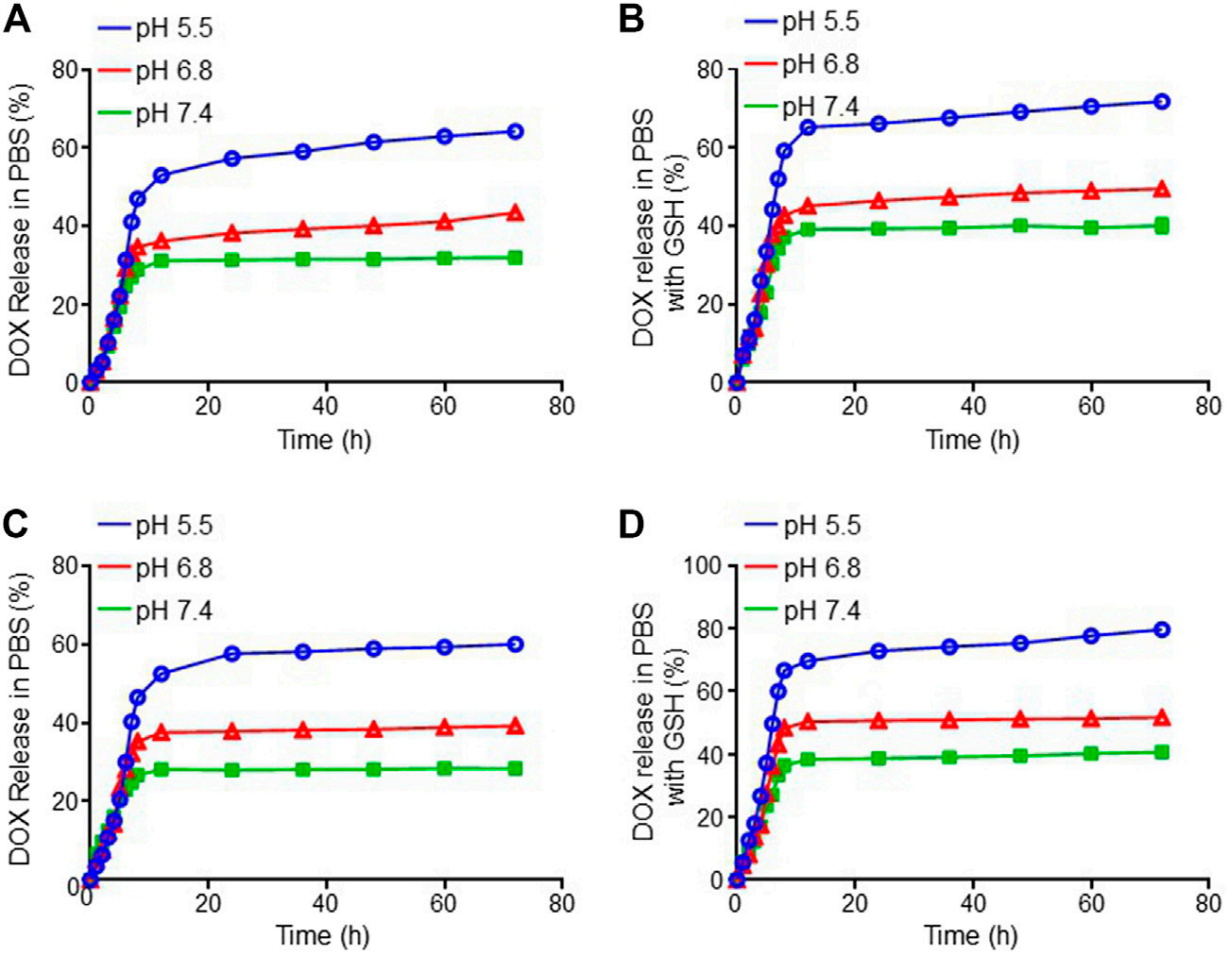
DOX release profiles of mNPs/DOX in **(A)** PBS and **(B)** PBS with GSH and STP-mNPs/DOX in **(C)** PBS and **(D)** PBS with GSH *in vitro* at 37°C, pH 5.5, 6.8, and 7.4, respectively. Data were presented as mean ± SD (*n* = 3).

The DOX release behaviors from both mNPs/DOX and STP-mNPs/DOX with or without GSH in different pH values *in vitro* were detected, respectively. As shown in Figure 2, a similar rapid release of DOX from mNPs/DOX and STP-mNPs/DOX is observed at 6 h, and DOX is released in a more steady pattern till 72 h subsequently. The data of DOX release behavior showed that both mNPs/DOX and STP-mNPs/DOX had a better release effect in the pH value 5.5 environments compared with pH values 6.8 and 7.4. The different pH values 5.5, 6.8, and 7.4 were similar to the pH values of the internal environment of tumor cells, the interstitial fluid, and the plasma, respectively. Therefore, the release of DOX from both mNPs/DOX and STP-mNPs/DOX is less in plasma than in the internal environment of tumor cells, showing that the nanoparticles prolonged the circulation time. More DOX was released from mNPs/DOX and STP-mNPs/DOX in the GSH environment than that in non-GSH environment, indicating that high levels of GSH in the tumor microenvironment could lead to more DOX release. The core-shell structure of STP-mNPs/DOX increases the stability of the nanoparticles in blood circulation and protects the loaded DOX. The disulfide bonds of STP-mNPs/DOX are ruptured within tumor tissues because of the rich GSH environment, and large amounts of DOX can be released. Due to the specific structure and the disulfide bonds, the developed STP-mNPs/DOX can deliver DOX to tumor cells in a targeted manner.

**FIGURE 3:**
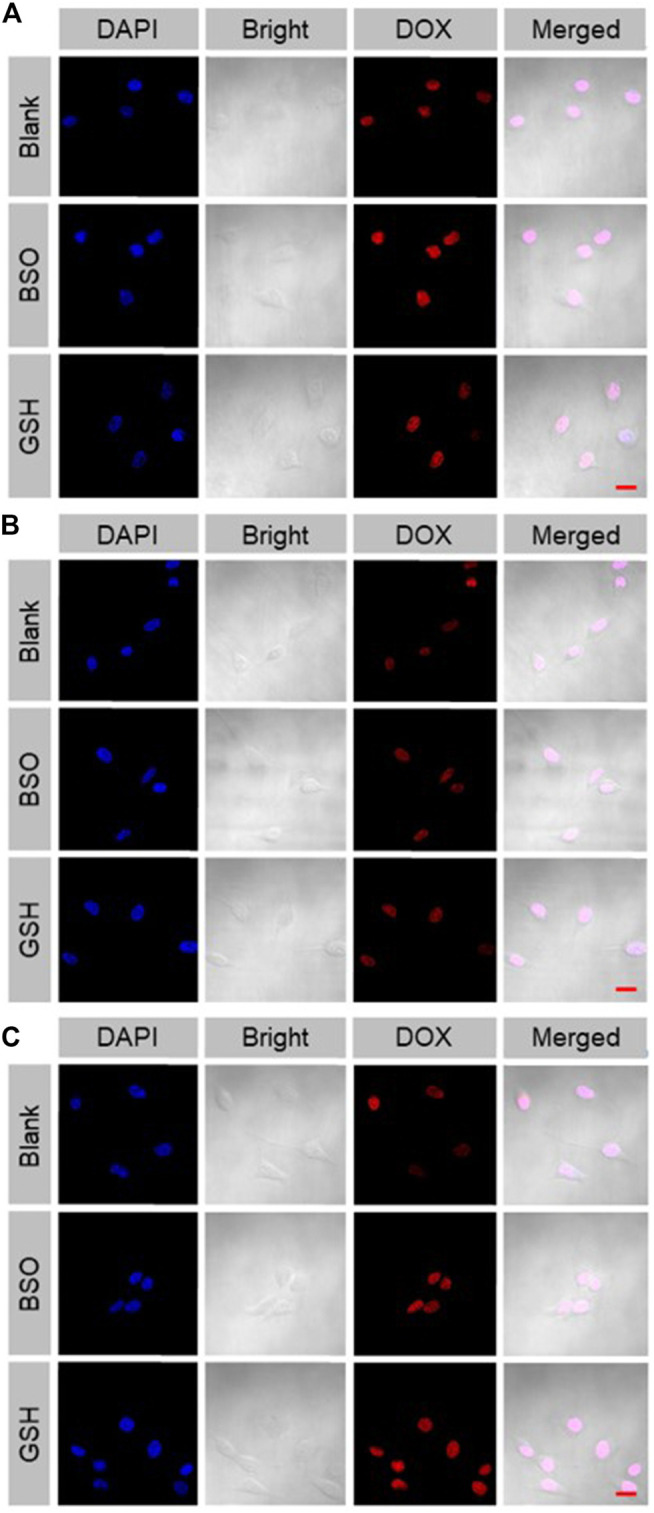
Cellular uptake and intracellular DOX release of **(A)** free DOX, **(B)** mNPs/DOX, and **(C)** STP-mNPs/DOX after incubation with CT26 cells for 2 h detected by CLSM. Scale bars = 20 μm.

**FIGURE 4:**
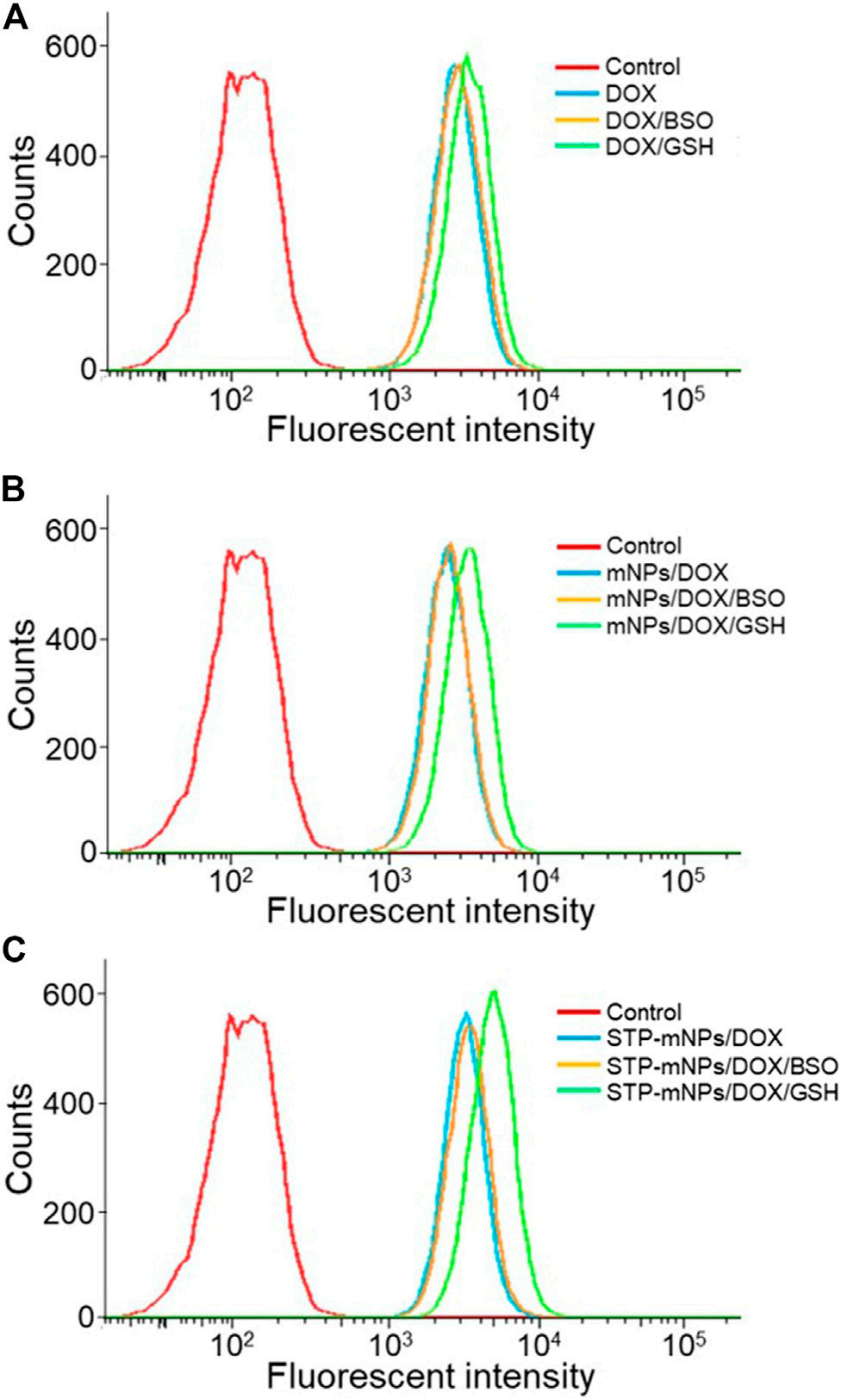
Cellular uptake and intracellular DOX release of DOX, mNPs/DOX, and STP-mNPs/DOX after incubation with CT26 cells for 2 h detected by FCM.

The cellular internalization and intracellular release of DOX were performed on the murine colon cancer cell line CT26. The CT26 cells were observed by CLSM after being treated with free DOX, mNPs/DOX, and STP-mNPs/DOX. DOX was directly used to measure the cellular internalization without any other additional markers due to its self-fluorescent characteristic, and the fluorescence intensity was directly proportional to the amount of internalized DOX (Hossain et al., 2013). As shown in Figure 3, the red fluorescence of DOX can be observed in the cell nuclei treated with free DOX, mNPs/DOX, and STP-mNPs/DOX, demonstrating the cellular internalization of mNPs/DOX and STP-mNPs/DOX and the intracellular DOX release of mNPs/DOX and STP-mNPs/DOX. For further evaluation, the cellular uptake of free DOX, mNPs/DOX, and STP-mNPs/DOX of the CT26 cells is analyzed with fluorescence-activated FCM (Figure 4). There is no significant difference in cellular internalization in the free DOX group after being treated for 2 h in PBS, PBS with BSO, or PBS with GSH. However, the cellular internalization or intracellular DOX release of mNPs/DOX and STP-mNPs/DOX in the PBS with GSH was more than PBS or PBS with BSO due to disulfide bonds in the mNPs/DOX and STP-mNPs/DOX possibly. BSO could inhibit the effects of GSH and mNPs/DOX and STP-mNPs/DOX are more stable after BSO treatment. However, GSH could increase the rupture of disulfide bonds, thus leading to more DOX being released. Therefore, fractures are more likely to occur in the GSH-induced reductive environment, thus accelerating DOX release from dissociated nanoparticles. These results further indicate that the rich GSH environment of tumor tissues can lead to large amounts of DOX release for tumor killing.

**FIGURE 5:**
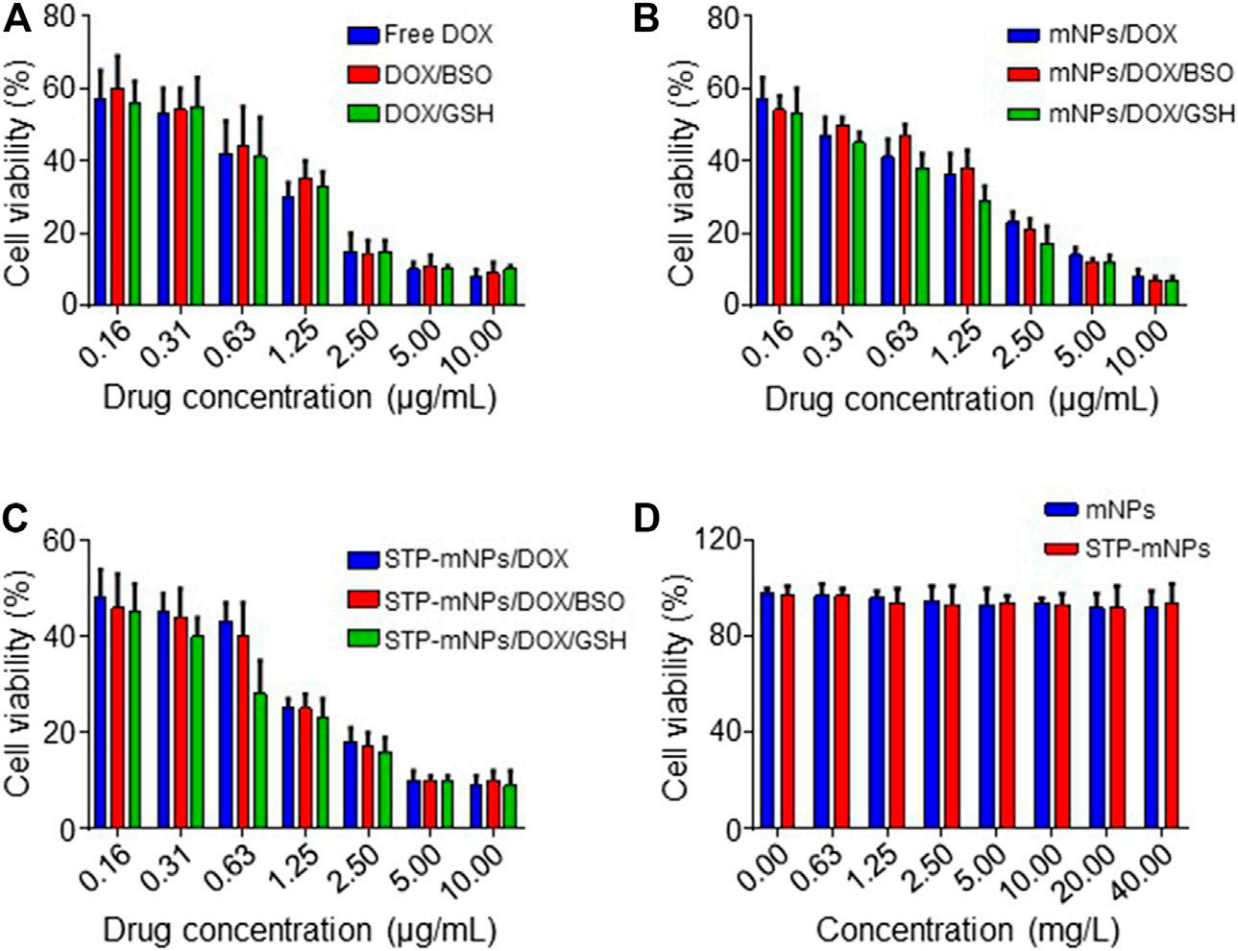
Cytotoxicities *in vitro* of free DOX, mNPs, STP-mNPs, mNPs/DOX, and STP-mNPs/DOX after incubation with CT26 cells for 48 h. Data were presented as mean ± SD (*n* = 3).

MTT assay was used to determine the cytotoxicity of free DOX, mNPs, STP-mNPs, mNPs/DOX, and STP-mNPs/DOX. CT26 cells were cultured in 96-well plates with PBS, BSO PBS solution, or GSH PBS solution, as previously described and treated with free DOX, mNPs/DOX, or STP-mNPs/DOX at an equal dosage of DOX, proceeding with MTT assay after 48 h. Figures 5A–C show that the viability of the CT26 cell decreases with the increase of DOX concentration and there is no significant difference between cell viability in the cells cultured with PBS, BSO PBS solution, or GSH PBS solution in the free DOX treated group. However, in mNPs/DOX and STP-mNPs/DOX treated group, the cell viability of cells cultured with GSH PBS solution was significantly lower than that of the cells cultured with PBS or BSO PBS solution, indicating that the cytotoxicity of mNPs/DOX and STP-mNPs/DOX was enhanced in reductive conditions, consistent with the previous result of DOX intracellular release. Figure 5D shows that mNPs and STP-mNPs have no toxic effect toward CT26 cells. The cell viability is still at a relatively high level with the increasing of mNPs and STP-mNPs concentrations. The synthesized mNPs and STP-mNPs indicated no cytotoxicity toward CT26 cells and can be safely applied for DOX delivery. STP-mNPs/DOX shows the best cytotoxicity toward CT26 cells under a rich GSH environment.

**FIGURE 6:**
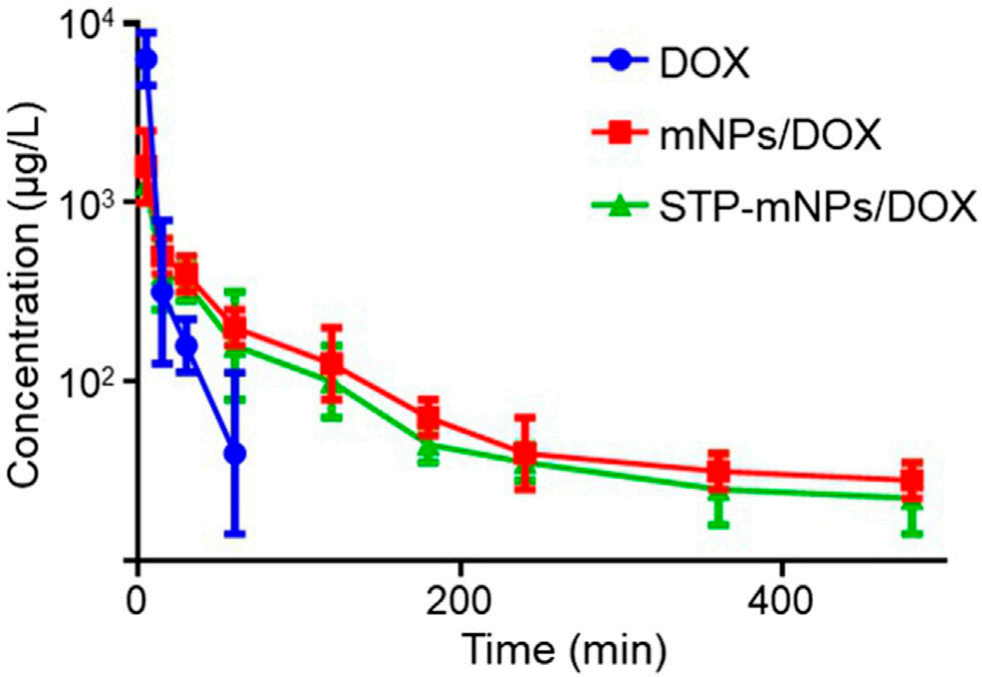
Pharmacokinetics of free DOX, mNPs/DOX, and STP-mNPs/DOX *in vivo*. Data were presented as mean ± SD (*n* = 3).

HPLC was used to evaluate the plasma pharmacokinetics of the free DOX, mNPs/DOX, and STP-mNPs/DOX (Zhu et al., 2010; Cho et al., 2012). Figure 6 shows that the DOX in plasma is cleared from blood circulation rapidly and can hardly be detected after being injected into the plasma for 60 min. In contrast, the DOX concentration of mNPs/DOX and STP-mNPs/DOX decreases relatively slower, showing that both mNPs/DOX and STP-mNPs/DOX increased the DOX stability in plasma in a slow-release mode. The results were consistent with previous DOX release profiles *in vitro*. The longer circulation time of mNPs/DOX and STP-mNPs/DOX could reduce the side effects of DOX to other major organs and increase the DOX accumulation in the tumor tissues (Qiu et al., 2020). The PEG shell of mNPs/DOX and STP-mNPs/DOX could seal DOX within the nanoparticles and protect the agent while passaging the blood flow. The disulfide bonds within the nanoparticles were stable in normal tissues but were sensitive to the microenvironment of tumor tissues with high levels of GSH. The suitable particle size of mNPs/DOX and STP-mNPs/DOX could also help maintain longer blood circulation time (Kolate et al., 2014). As mentioned above, the diameter of STP-mNPs/DOX (90.74 ± 4.65 nm) enables the effective delivery of DOX to tumor cells without being captured by the reticuloendothelial system or eliminated from plasma rapidly (Peer et al., 2007; Wang et al., 2012).

**FIGURE 7:**
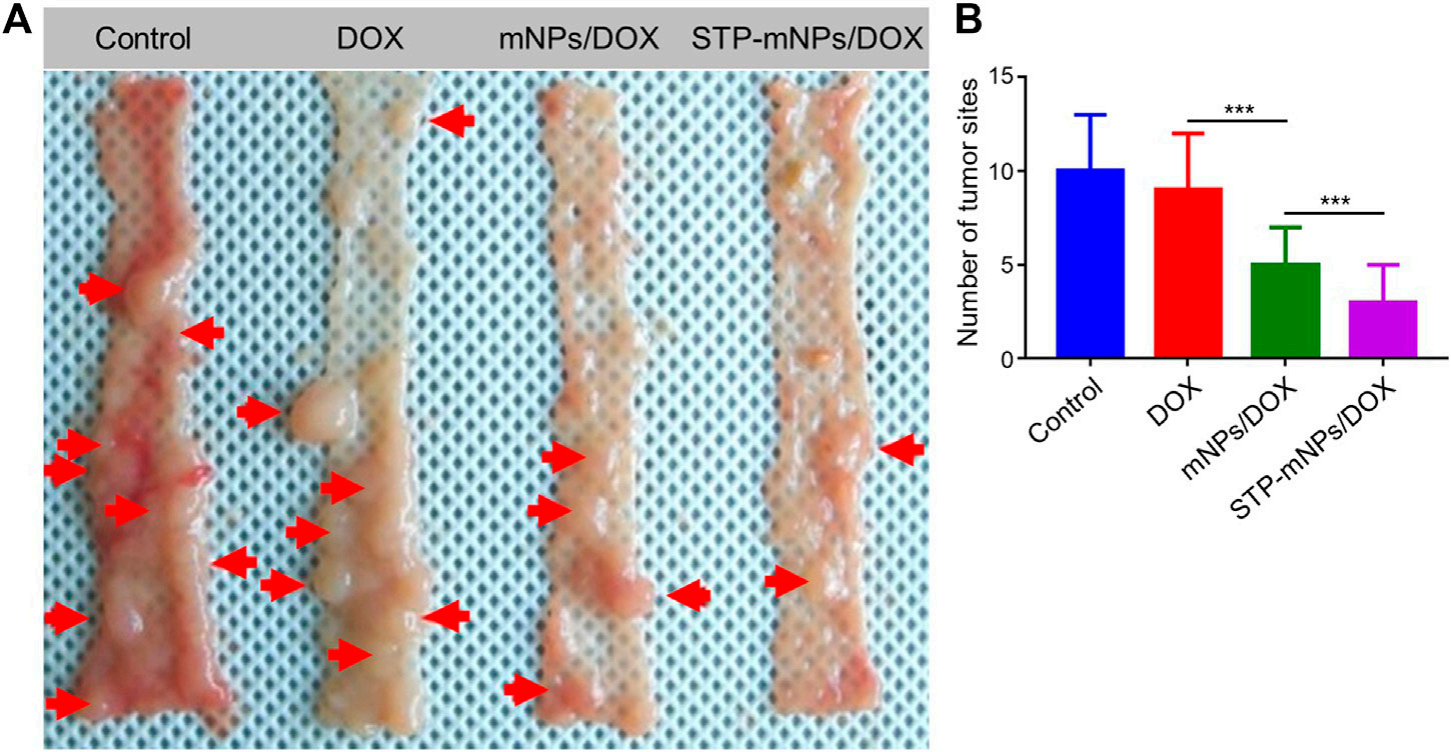
The treatment efficacy of STP-mNPs/DOX toward primary orthotopic colon cancer in mice. **(A)** The tumor and colon were dissected from mice treated with free DOX, mNPs, or STP-mNPs/DOX, with PBS as control. The red arrows pointed to tumors that are larger than 2 mm in diameter. **(B)** The number of tumor sites which are larger than 2 mm in diameter. Data were presented as mean ± SD (*n* = 6, ***p < 0.001).

**FIGURE 8:**
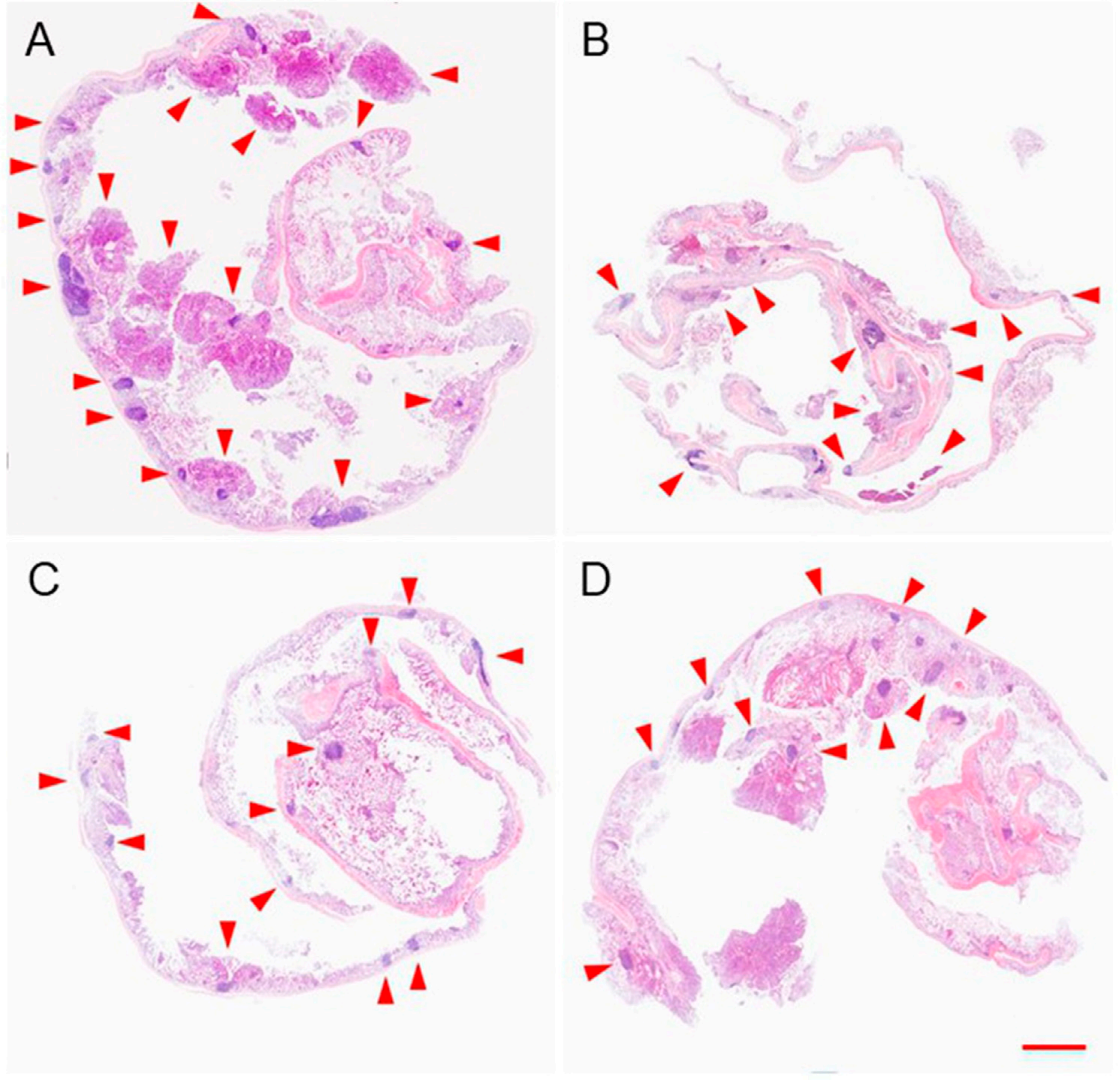
Colon and tumor sections of mice after treatment of **(A)** PBS, **(B)** free DOX, **(C)** mNPs/DOX, or **(D)** STP-mNPs/DOX were stained by H&E. The red arrows pointed to tumors. Scale bar = 5 mm.

DMH was used as the inducer to establish the colon cancer *in situ* tumor animal models (Samanta et al., 2008; Baskar et al., 2012). Hematochezia occurred in many Balb/C mice, and ascites occurred in only two mice during the drug-induced process caused by colon cancer formed in the intestines possibly. The growth or burst of colon cancer could cause bleeding within the colonic cavity. The DMH-induced process lasted for 4 months. After the last intraperitoneal injection, mice were randomly divided into PBS, free DOX, mNPs/DOX, and STP-mNPs/DOX groups, respectively, and administred with a DOX dose of 5 mg kg^−1^ through intravenous injection every 4 days for a total of six injections. Free DOX and PBS were administred as positive and negative controls, respectively. The mice were sacrificed, and the colon tissues were dissected and washed out in deionized water after the treatment process. Figure 7A shows the photographed results of the flattened colons. Tumor sites with diameters larger than 2 mm were counted. The amount of the tumor of the STP-mNPs/DOX treated mouse is significantly less than the others (Figure 7B), indicating that the STP-mNPs/DOX has the best treatment effect. Subsequently, the flattened colons were curled up along the long axis and fixed with 4% paraformaldehyde. The winding colon was sliced along the winding direction and the tissues were H&E stained and photographed under the microscope. Figure 8 shows that the nucleus gathered zones are tumor sections. The amount of the tumor of STP-mNPs/DOX treated mice was significantly less than the other mice on the micro-level. The accumulation of nanoparticles at the tumor site was enhanced due to the EPR effect (Kobayashi et al., 2013) and tumor-targeting effect (Qiu et al., 2020), leading to the enhanced antitumor efficacy of STP-mNPs/DOX. The disulfide bonds made STP-mNPs/DOX release DOX more efficiently in the intercellular environment.

**FIGURE 9:**
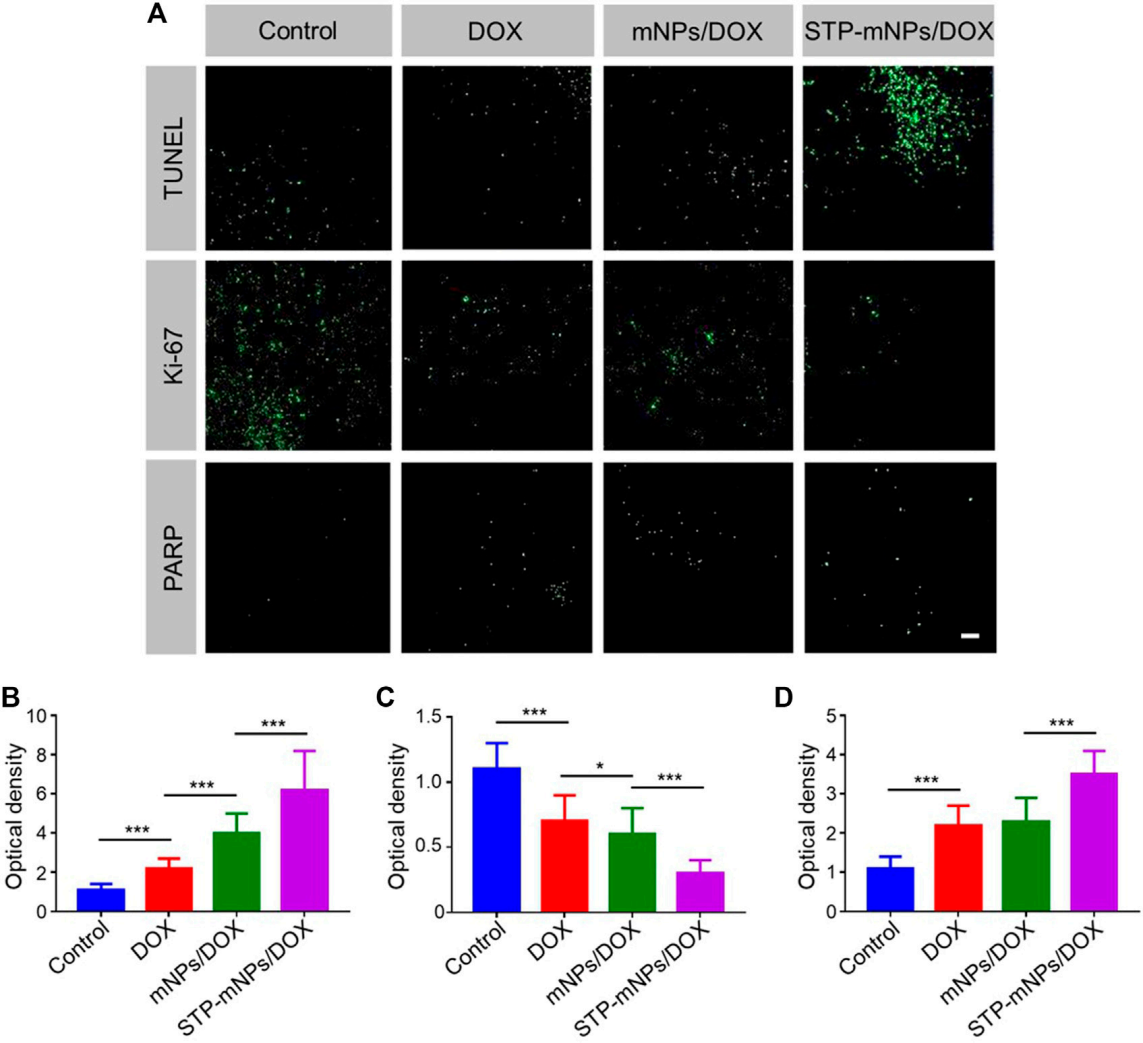
Immunofluorescence analysis of TUNEL, Ki-67, and PARP in Control, DOX, mNPs/DOX, and STP-mNPs/DOX groups. **(A)** CLSM of the immunofluorescence images in different groups. Semiquantitative analysis of **(B)** TUNEL, **(C)** Ki-67, and **(D)** PARP in different groups. Data were presented as mean ± SD (*n* = 3, *p < 0.05 and ***p < 0.001). Scale bar = 50 μm.

To further evaluate the antitumor efficacy of DOX, mNPs/DOX, and STP-mNPs/DOX, the colons were collected after the mice dead and sectioned for TUNEL assay. The DNA of tumor cells was fractured caused by chemotherapy drugs, and the fragmentation of DNA could be dyed with green fluorescence by a fluorescein isothiocyanate marked TUNEL kit. Figure 9A shows that the flake and punctiform green fluorescent signals are observed in DOX-loaded nanoparticles or free DOX treated tumor, and the green fluorescence signal is relative to the tumor cell apoptosis. The images are taken from the junction of the tumor and normal tissue, showing that there was no visible fluorescence signal in the location of normal tissue. However, in the tumor location, more fluorescence signal was observed in the tumor of the STP-mNPs/DOX treated group than the other groups, indicating that more apoptosis took place in the STP-mNPs/DOX treated tumor. Therefore, the antitumor efficacy of STP-mNPs/DOX was better than that of the others. The results were also confirmed by semiquantitative analysis. The fluorescence intensity of the control group was defined as “1,” and the optical densities of the other groups were defined as the ratio of the sample group and the control group. Figure 9B shows that the STP-mNPs/DOX treated group has the most TUNEL expression compared to the other groups. The Ki-67 and PARP assays were processed in the tumor area to confirm the tumor apoptosis further. Figure 9A shows different results because the Ki-67 and PARP assays are a pair of opposite markers. The tumor treated with STP-mNPs/DOX shows the lowest fluorescent signal in the Ki-67 assay, reflecting the lowest tumor proliferation ability in STP-mNPs/DOX group. The STP-mNPs/DOX group also showed the highest fluorescent signal in PARP assay, reflecting tumor apoptosis. The results indicate that STP-mNPs/DOX can effectively promote tumor apoptosis and inhibit tumor proliferation, consistent with Figure 9A. Figures 9C,D also show the least and most fluorescent intensities of TUNEL and PARP in the STP-mNPs/DOX group, respectively. The histopathological studies show that STP-mNPs/DOX can significantly prevent the progression of colon cancer by inhibiting tumor proliferation and increasing tumor apoptosis.

**FIGURE 10:**
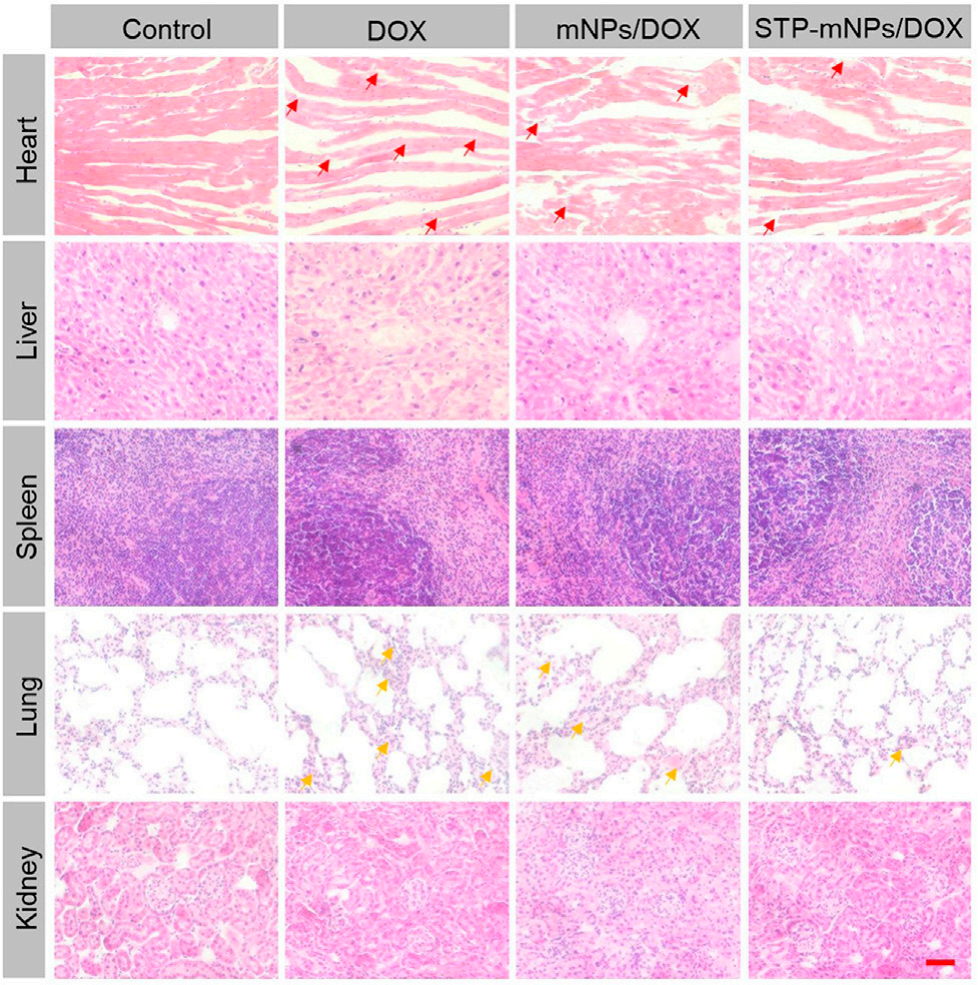
*In vivo* histological (H&E) analyses of major organs (heart, liver, spleen, lung, and kidney) sections form *in situ* tumor model mice after treatment with free DOX, mNPs/DOX, STP-mNPs/DOX, or PBS (as control). Red arrows indicate myocardial injuries and yellow arrows indicate lung injuries. Scale bar = 100 μm.

For antitumor drugs, security assessments were crucial in the clinical application (Ding et al., 2011). The major organs (heart, liver, spleen, lung, and kidney) were dissected from mice after death to evaluate the safety of STP-mNPs/DOX. The organs were sliced and stained by H&E. The stained sections were photographed for histopathological analyses. As shown in Figure 10, the images of major organs of each group are exhibited. The images of organs of the control group show that no significant tissue damage occurs, and every organ has a relatively normal histological structure. However, other groups treated with DOX formulation showed different degrees of myocardial injuries, such as myocardial cell edema, irregular arrangement of the myocardial cell, and inflammatory cell infiltration. In the meantime, pathological lung changes could be observed in the DOX formulation treated group, such as thickening and blocking alveolar walls. Moreover, the organ damage level of the STP-mNPs/DOX treated group was lower than that of mNPs/DOX or free DOX treated group. Nevertheless, all the visible changes were slight, and no significant morphological changes could be detected, showing the high biosecurity of STP-mNPs/DOX.

## Conclusion

DOX-loaded tumor-targeting peptide-decorated polypeptide nanoparticles were synthesized to treat DMH-induced *in situ* colon cancer in mice. *In vitro* experiments of STP-mNPs/DOX showed appropriate size for tumor tissue accumulation, improved DOX release and internalization, and enhanced antitumor efficacy against CT26 cells. *In vivo* experiments in mice with the orthotopic colon cancer model demonstrated that STP-mNPs/DOX possess better antitumor efficacy than mNPs/DOX and free DOX. The synthesized STP-mNPs/DOX shows appropriate properties as one of the DDSs and exhibits good antitumor properties after encapsulating DOX. Therefore, STP-mNPs can be applied in delivering a series of small-molecule chemotherapeutic drugs for cancer therapy.

## Data Availability

The raw data supporting the conclusion of this article will be made available by the authors, without undue reservation.
