# Supplementary figures and images for: Doxorubicin-Loaded Tumor-Targeting Peptide-Decorated Polypeptide Nanoparticles for Treating Primary Orthotopic Colon Cancer
Source: Front Pharmacol. 2021 Oct 15;12:744811. doi: 10.3389/fphar.2021.744811 (PMC8554036; doi:10.3389/fphar.2021.744811)

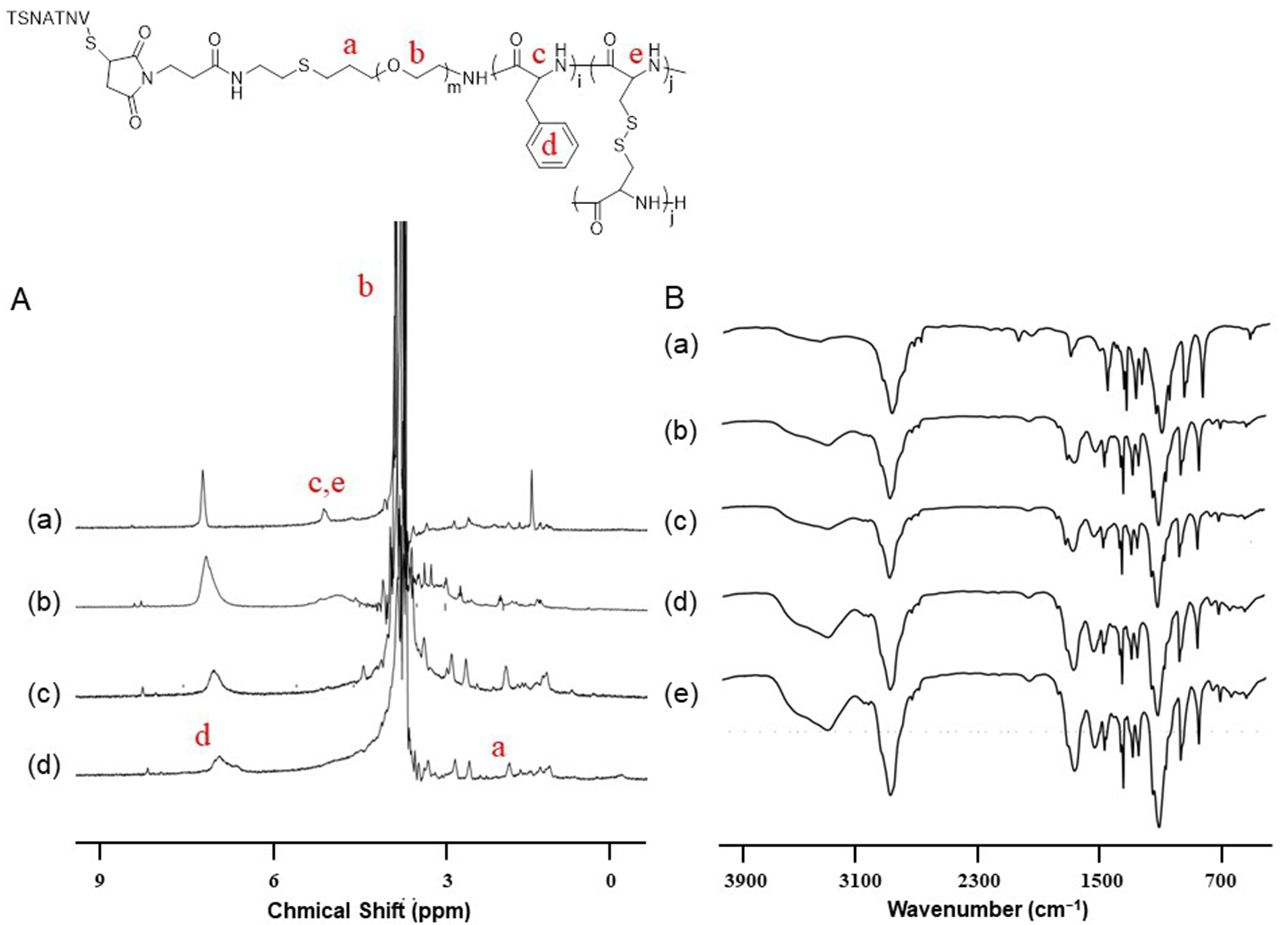

Supplement: Supplementary file 1 [file Image1.JPEG]
